# Rapid-Onset Obesity with Hypoventilation, Hypothalamic, Autonomic Dysregulation, and Neuroendocrine Tumors (ROHHADNET) Syndrome: A Systematic Review

**DOI:** 10.1155/2018/1250721

**Published:** 2018-11-21

**Authors:** Jiwon M. Lee, Jaewon Shin, Sol Kim, Heon Yung Gee, Joon Suk Lee, Do Hyeon Cha, John Hoon Rim, Se-Jin Park, Ji Hong Kim, Ahmet Uçar, Andreas Kronbichler, Keum Hwa Lee, Jae Il Shin

**Affiliations:** ^1^Department of Pediatrics, Chungnam National University Hospital, Daejeon, Republic of Korea; ^2^Department of Pediatrics, Yonsei University College of Medicine, Seoul, Republic of Korea; ^3^Yonsei University Wonju College of Medicine, Seoul, Republic of Korea; ^4^Department of Pharmacology, Yonsei University College of Medicine, Seoul, Republic of Korea; ^5^Department of Pediatrics, Geoje Children's Hospital, Ajou University School of Medicine, Geoje, Republic of Korea; ^6^Department of Pediatric Endocrinology and Diabetes, Health Sciences University, Sisli Hamidiye Etfal Education & Research Hospital, Istanbul, Turkey; ^7^Department of Internal Medicine IV (Nephrology and Hypertension), Medical University Innsbruck, Innsbruck, Austria; ^8^Division of Pediatric Nephrology, Severance Children's Hospital, Seoul, Republic of Korea; ^9^Institute of Kidney Disease Research, Yonsei University College of Medicine, Seoul, Republic of Korea

## Abstract

**Background and Aim:**

ROHHADNET (rapid-onset obesity with hypoventilation, hypothalamic, autonomic dysregulation, neuroendocrine tumor) syndrome is a rare disease with grave outcome. Although early recognition is essential, prompt diagnosis may be challenging due to its extreme rarity. This study aimed to systematically review its clinical manifestation and to identify genetic causes.

**Materials and Methods:**

We firstly conducted a systematic review on ROHHAD/NET. Electronic databases were searched using related terms. We secondly performed whole exome sequencing (WES) and examined copy number variation (CNV) in two patients to identify genetic causes.

**Results:**

In total, 46 eligible studies including 158 patients were included. There were 36 case reports available for individual patient data (IPD; 48 patients, 23 ROHHAD, and 25 ROHHADNET) and 10 case series available for aggregate patient data (APD; 110 patients, 71 ROHHAD, and 39 ROHHADNET). The median age at onset calculated from IPD was 4 years. Gender information was available in 100 patients (40 from IPD and 60 from APD) in which 65 females and 35 males were showing female preponderance. Earliest manifestation was rapid obesity, followed by hypothalamic symptoms. Most common types of neuroendocrine tumors were ganglioneuromas. Patients frequently had dysnatremia and hyperprolactinemia. Two patients were available for WES. Rare variants were identified in* PIK3R3*,* SPTBN5,* and* PCF11* in one patient and* SRMS*,* ZNF83,* and* KMT2B* in another patient, respectively. However, there was no surviving variant shared by the two patients after filtering.

**Conclusions:**

This study systematically reviewed the phenotype of ROHHAD/NET aiming to help early recognition and reducing morbidity. The link of variants identified in the present WES requires further investigation.

## 1. Introduction

Rapid-onset obesity with hypoventilation, hypothalamic, autonomic dysregulation (ROHHAD) syndrome is a rare disorder of respiratory failure and autonomic dysregulation with endocrine abnormalities [1]. The suffix -NET was later added to describe a subset of patients with ROHHAD who were found with neuroendocrine tumors (NET) as ROHHADNET [2].

ROHHAD or ROHHADNET may mimic genetic obesity syndromes and present with hypothalamic-pituitary dysfunctions which are not fully investigated [3]. Since the central respiratory control becomes progressively impaired in the patients, the outcome is often fatal and associated with cardiopulmonary arrest [4]. Prompt diagnosis based on early recognition is essential to provide timely respiratory support and to minimize morbidity and mortality. We thereby sought to systematically review the clinical manifestation, laboratory profiles, and treatment strategies of patients with ROHHAD/NET to help understanding and managing the disease. In addition, we performed whole exome sequencing (WES) in 2 patients with ROHHADNET in the attempt to identify the genetic causes.

## 2. Methods

### 2.1. Search Methods

**Figure 1:**
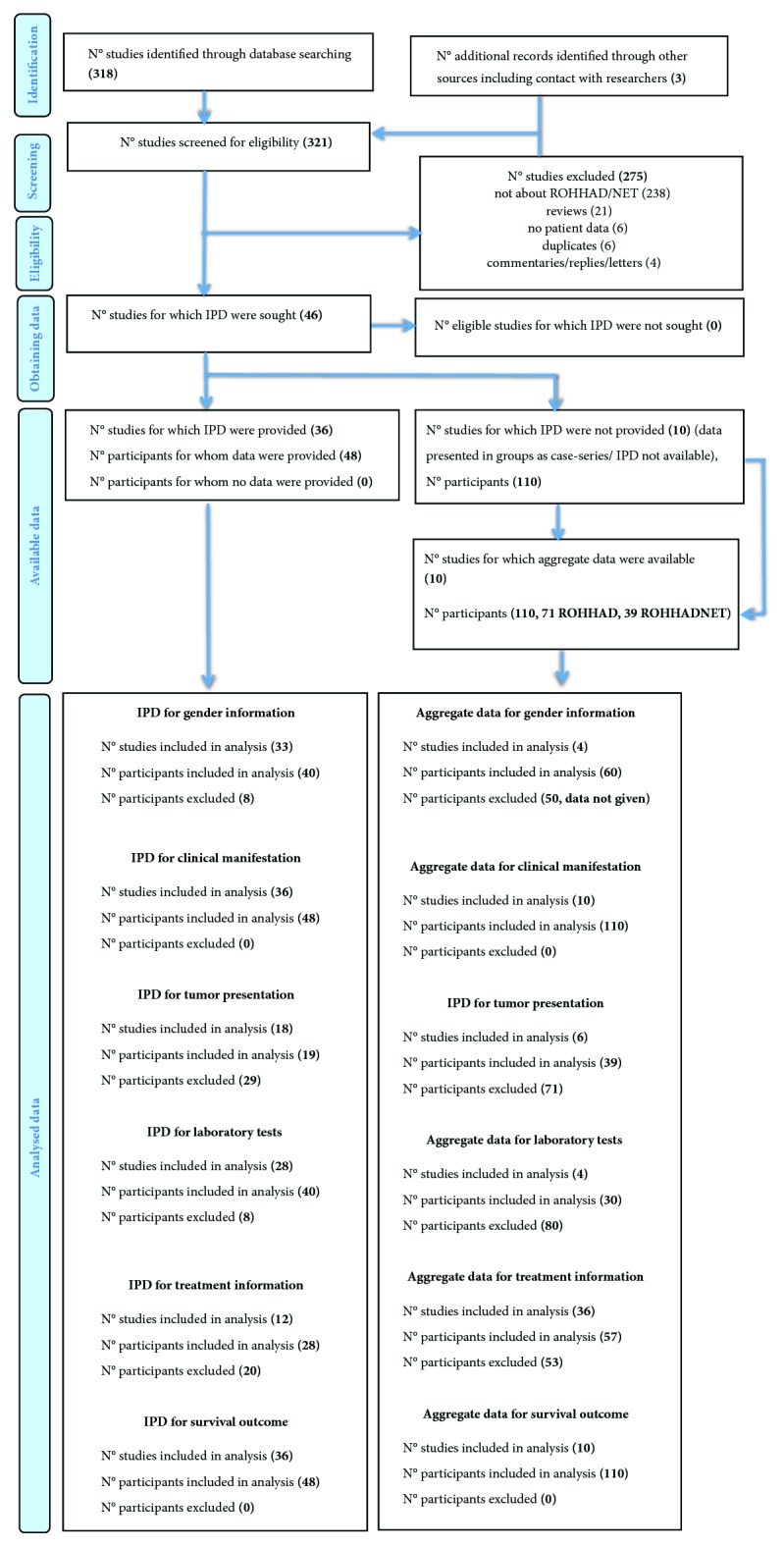
PRISMA IPD flow diagram.

We conducted a systematic review of the medical literature to identify all published cases of ROHHAD and/or -NET using the online databases of MEDLINE/PubMed, EMBASE, and Google Scholar, until July 7th, 2018. There were no language restrictions; non-English language articles were translated and included. The broad search query was designed to include “ROHHADNET” OR “ROHHAD”; OR “obesity” AND two of the following terms; “hypoventilation” OR “hypothalamic” OR “autonomic” OR “tumor” OR “neural crest tumor” OR “neuroendocrine tumor.” We reviewed the titles, abstracts, and full texts adhering to the Preferred Reporting Items for Systematic Reviews and Meta-analyses (PRISMA) individual patient data (IPD) guidelines (Figure 1; Supplementary Table S1) [5].

### 2.2. Eligibility Criteria

The basic criteria for consideration of the diagnosis of ROHHAD had been published by Ize-Ludlow et al. [6]. The criteria briefly included the following: (1) onset of rapid and extreme weight gain after an age of 1.5 years (typically 2–7 years) in a previously nonobese and seemingly normal child, (2) evidence of hypothalamic dysfunction, (3) alveolar hypoventilation, and (4) features of autonomic dysregulation. We collected published case reports and case series which contained data on clinical manifestations fulfilling the criteria of ROHHAD/NET. Due to the extreme paucity of data, congress abstracts were also included. All cases from the literature were included as applicable.

### 2.3. Exclusion Criteria

Duplicates, letters, commentaries, or replies were excluded. Original articles not containing patient data, such a review articles, were also excluded.

### 2.4. Selection of Studies

Two reviewers (J.M.L, and S.K.) working independently considered the potential eligibility of each abstract and title that resulted from the initial search. The full-text versions of the eligible studies were reviewed. Disagreements were harmonized by consensus and, if not possible, through arbitration by a third reviewer (J.I.S.).

### 2.5. Data Extraction

Data were extracted from all of the case reports and case series which were included in the systematic review. Demographic information included age, gender, and ethnicity. Clinical manifestation included presence of symptoms, such as hypothalamic dysfunction, hypoventilation, autonomic dysregulation, neuroendocrine tumors, and neurologic or other remarkable reports. Data regarding the laboratory findings, management strategy, and clinical outcomes were also examined.

### 2.6. DNA Preparation, Whole Exome Sequencing, Sequence Alignment, and Variant Calling

This study was approved by the institutional review board of the Severance Hospital, Yonsei University Health System** (IRB No.2017-2991-001)**. There were two patients with ROHHADNET with available samples, Case 1 [7] and Case 2 [8] (Supplementary Table S2). After obtaining informed consent, whole blood (3 ml) was collected from the two individuals with ROHHADNET. Genomic DNA was extracted using RBC Lysis Solution, Cell Lysis Solution, and Protein Precipitation Solution (iNtRon Biotechnology, Inc). Whole exome capture was performed using the Agilent SureSelect V5 enrichment capture kit (Agilent Technologies). The enriched library was then sequenced using the HiSeq 2500 sequencing system (Illumina; 101-base paired-end sequencing). Image analysis and base calling were performed with the Pipeline software (Illumina) using default parameters. Sequence reads were mapped to the human reference genome assembly (GRCh37/hg19) using the CLC Genomic Workbench (version 9.0.1) software (QIAGEN). Mapping was performed using the “Map Reads to Reference” function of the CLC Genomic Workbench software with the following settings: mismatch cost, 2; insertion cost, 3; deletion cost, 3; length fraction, 0.5; similarity fraction, 0.9; and map to nonspecific reads, random. Nonspecific reads were ignored for count and coverage. All variants with a minimum coverage of 2 were called using the “Basic Variant Caller” function of the CLC Genomic Workbench and annotated.

### 2.7. Filtering and Evaluation of Variants

Whole exome sequencing was analyzed as previously described [9]. Briefly, variants with minor allele frequencies >1% in the single nucleotide polymorphism (dbSNP; version 138) or 1000 genomes (2504 individuals; phase 3 data) databases were excluded. In the second step, variants present in the homozygous or hemizygous state in 59 healthy individuals without ROHHAD syndrome (internal control WES data) were excluded. In step 3, synonymous variants and intronic variants not located within splice site regions were excluded. In step 4, a recessive inheritance pattern was assumed on the basis of the pedigree of affected individuals. Therefore, homozygous and biallelic compound heterozygous variants were retained, while single heterozygous variants were excluded from further evaluation. In Case 1 who was a male, hemizygous variants were also considered. De novo variants could not be evaluated because parental DNAs were not available. In the final step, the remaining variants were ranked based on conservation of the mutated amino acid residue across species and their probable impact on the function of the encoded protein. The remaining variants were confirmed in the original participant DNA samples by Sanger sequencing.

### 2.8. Copy Number Variant (CNV) Analysis

Analysis of CNV was performed using the paired-end WES data using the EXCAVATOR version 2.2 [10] and ExomeDepth version 1.1.10 tools [11] with default settings. The GRCh37/hg19 database was used as the reference assembly for calculation of GC content. The WES dataset of 11 internal control subjects was compared with that of the study participants. Copy number variations at specific target regions were estimated according to different CNV detection algorithms using the Agilent SureSelect V5 kit.

## 3. Results

In total, 321 articles were identified using electronic and manual search methods (Figure 1). After serially reviewing the titles, abstracts, and full texts, 46 eligible studies including 158 patients were included. Among them, there were 36 case reports available for individual patient data (IPD; 48 patients, 23 ROHHAD and 25 ROHHADNET) [3, 4, 7, 8, 12–43]. The remaining ten studies were reporting patients in groups or cohorts and were therefore available for aggregate patient data (APD; 110 patients, 71 ROHHAD and 39 ROHHADNET) [6, 44–50].

Data regarding gender were available in 100 patients (40 from IPD and 60 from APD). There were 65 females and 35 males showing female preponderance, and female to male ratio was 1.9 to 1. Aside from gender, most of clinical information was extracted from 36 case reports where IPD were available. Limited information was retrievable from 10 studies with APD. Detailed profiles of the studies and patients' data are presented in Tables 1 and 2.

### 3.1. Individual Patient Data (IPD) from Case Reports

There were 48 patients in the 36 case reports, in which 100% were pediatric cases. The median age at the time of diagnosis was 4.0 years (range, 1-15). Twelve patients (12/40, 30 %) were boys, 28 (28/40, 70%) were girls, and no information could be retrieved in the 8 remainders. Female to male ratio from IPD was 2.3 to 1.

#### 3.1.1. Clinical Presentation

The most common presentation of patients with ROHHAD/NET was rapid obesity and hypothalamic dysfunction found in 40 cases (83%) respectively, followed by hypoventilation reported in 36 cases (75%). Hypothalamic dysfunction presented in various forms of endocrine disorder, such as growth hormone deficiency (25%), diabetes insipidus (19%), and central precocious puberty (15%). Hypoventilation most commonly presented as obstructive sleep apnea (44%). For symptoms of autonomic dysregulation, ophthalmologic abnormality such as blurred vision was most commonly reported (25%), followed by altered pain perception (13%) and gastrointestinal dysmotility (13%). Excessive sweating was noted in 10% of the patients. Behavioral change was a common (60%) form of cognitive dysfunction, and the symptoms included mood changes, fatigue, social withdrawal, poor school performance, and intellectual disability. Other neurologic manifestations majorly included seizures, altered consciousness, sleep disturbance, and developmental delay. The clinical presentations of the patients are summarized in Table 3.

#### 3.1.2. Laboratory Findings

In 13 patients who had available datasets, all had hypoxemia at initial presentation and hypercapnia was also dominant (14/15, 93%; Table 4). Dysnatremia was accompanied in most of the patients (30/31, 97%): 25 hypernatremia and 5 hyponatremias. Hyperprolactinemia (27/28, 96%), decreased IGF-1 level (12/16, 75%), and hypothyroidism (18/30, 60%) were also common.

#### 3.1.3. Treatment Strategies and Survival

At the time of diagnosis, high proportion of patients (21/48, 44%) required respiratory support: mechanical ventilation in 20 (42%) cases and tracheostomy in 6 (13%) cases (Table 5). Six of the 44 (14%) patients were treated with steroids, while other immunosuppressive measures including rituximab and/or cyclophosphamide were administered in 7 cases (7/48, 14%). There were 4 deaths (3 sudden cardiac arrests and 1 multiorgan failure after sepsis) out of the 48 cases (Table 1).

#### 3.1.4. Tumor Presentation

Out of 48 patients, twenty-five had neuroendocrine tumors (52.1%). The features of the tumors are described in Table 6. The most common type was ganglioneuromas: 15 ganglioneuromas (60%), 9 ganglioneuroblastomas (36%), and 1 hamartoma with neural tissue (2%). Although the lesions usually presented as intra-abdominal mass, 2 cases with mediastinal masses were reported.

### 3.2. Aggregate Patient Data (APD)

The 10 studies with APD included 110 patients (Figure 1; Table 2). Although limited data were available regarding age, all of the reported were pediatric cases. Sixty patients were available for gender information: 23 males (38%) and 37 females (62%). Female predilection was consistently noted. Rapid-onset obesity was observed in 65% (71/110) of the patients. Hypoventilation was reported in 51/110 (46%) patients, 63% of them (32/51) presented with sleep apnea, supporting the findings from IPD. Autonomic dysfunction was reported in 80/106 (75%) patients and behavioral changes were observed in 40/110 (36%). There were 46/110 (42%) patients who had neuroendocrine tumors and ganglioneuroma was the most common type as in IPD (12/46; the remaining 34 were not available for histology). In line with the IPD results, dysnatremia was the most commonly observed electrolyte imbalance (21/27, 78%). Information regarding treatment strategies was available in 51 patients and 100% of them eventually received artificial ventilation. There were 12 deaths (9 sudden cardiac arrests and 2 not available for cause of death) out of the 110 patients. The frequencies and characteristics of clinical manifestation generally conformed to those from IPD.

### 3.3. Next Generation Sequencing

We described previously reported human candidate genes [6, 12, 51–54] for ROHHAD/NET in Table 7. None of these, however, have been identified in the patient cases to date. In our study, there were two ROHHADNET patients with available samples for whole exome sequencing: Case 1, a 15-year-old Korean boy [7]; and Case 2, a 5-year-old Turkish girl [8]. Details with regard to these two patients are briefed in Supplementary Table S2. Currently, there is no known genetic cause for ROHHAD or ROHHADNET [55]. To identify genetic variants related to ROHHAD syndrome, we performed WES for Case 1 and Case 2. Since ROHHAD syndrome in these individuals was sporadic and had childhood onset, we assumed the following inheritance patterns: (1) biallelic variants in recessive genes and (2) hemizygous variants in X-chromosome genes in Case 1. Variant filtering reduced the number of candidate genes to five in Case 1 and three in Case 2, respectively, as outlined in Supplementary Table S3. In Case 1, variant filtering was begun with 188,415 variants from the normal reference sequence. This number was reduced to 1,914 upon exclusion of homozygous and hemizygous variants in healthy domestic individuals, common variants (minor allele frequencies >1% in public databases), and synonymous variants. Upon considering only those genes with hemizygous variants or more than two variants in the same gene, the number of variants was further reduced to 50 variants (14 genes). Exclusion of artefacts by direct inspection of sequence alignment and exclusion of variants with minor allele frequencies < 0.005 in public databases left six variants in three candidate genes—*PIK3R3*,* SPTBN5,* and* PCF11* (Supplementary Table S4). These variants were predicted likelihood to be deleterious for the function of the encoded protein in some prediction tools and* PIK3R3, SPTBN5,* and* PCF11* are not linked to any disease phenotype in human yet. The WES of Case 2 was analyzed in the same manner to identify candidate variants (Supplementary Table S4), but none of them overlapped with variants identified in Case 1;* SRMS* and* ZNF4* were not linked to any disease phenotypes, whereas mutations in* KMT2B*, which encodes lysine-specific methyltransferase 2B, cause childhood-onset dystonia [56]. All variants were confirmed by Sanger sequencing of the DNA of the affected individuals.

In addition, we analyzed CNVs; has been previously abbreviated using WES in Case 1 and Case 2. The CNVs detected by both EXCAVATOR and ExomeDepth tools were 38 in Case 1 and 48 in Case 2, respectively. We specifically focused on deletion or duplication of alleles in an AR pattern; however, there was no surviving CNV upon manual inspection of WES data.

## 4. Discussion

ROHHAD/NET is a rare disease and differential diagnosis from other obesity syndromes or neuroendocrine disorders requires clinical suspicion based on its phenotype. The genetic basis of this syndrome is still unknown.

The first part of this study is a systematic review on phenotypes of ROHHAD/NET involving 46 studies with 158 patients. Clinical manifestation, laboratory findings, tumor characteristics, and patient courses were reviewed. The results showed that it has a pediatric onset and it is noteworthy that no adult case has been reported to date. There was a female preponderance, with the girls being twice as often affected than the boys, consistently in both IPD and APD. This finding is in contrast to what has been reported on acquired sleep disorders with a 2:1 predominance of males in the reported frequency of obstructive sleep apnea [57]. Rapid obesity may often be the first recognizable sign, since other endocrine dysfunctions are gradually present. The results implicated that common endocrine disorders such as hypothyroidism or precocious puberty may be early signs for recognition. In addition, it has been reported that one of the major effects of hypothyroidism is its influence on the central ventilatory control and that both hypoxic and hypercapnic ventilatory impairment are significantly present in untreated thyroid insufficiency [58]. Such impaired ventilatory responses are thought to be related to the decrease in oxygen consumption associated with hypothyroidism [59]. In that, it is tempting to speculate that disturbance of thyroid function may be in part responsible for respiratory distress in patients with ROHHADNET. Electrolyte imbalance, especially dysnatremia, was present in a majority of the patients, requiring attention. Impaired water balancing condition such as polydipsia or diabetes insipidus due to hypothalamic dysfunction may have caused dysnatremia. Ganglioneuromas were the most common type of accompanied tumor and may presented not only as abdominal but also as mediastinal masses. We therefore suggest that suspected patients take both thoracic and abdominal imaging to screen for tumors. As ROHHAD/NET involves progressive impairment of the respiratory center, we observed that artificial ventilation was commonly initiated from the first place. Cardiac arrest probably due to preceding respiratory arrest was the major cause of deaths in these patients. We noted that all of the patients were already exposed to hypoxemia at the time of diagnosis. We believe that earlier recognition and timely application of pressure supporting devices during sleep may improve the quality of life and prevent sudden death.

The second part of this study was a WES which attempted to identify the genetic basis of ROHHAD/NET. It has been noted that central hypoventilation syndrome (CHS) resulting from* PHOX2B* mutations is associated with tumors of neural crest origin (neuroblastoma, ganglioneuroblastoma, and ganglioneuroma) in approximately 6% of cases [59]. However, the association of ROHHADNET and* PHOX2B* mutations has not been identified. Recently, several studies have made progress in investigating genetic basis of ROHHAD/NET (Table 7). Thaker et al.[12] identified a* de novo* retinoic acid-induced 1 (*RAI*1) gene mutation in a child with ROHHAD and proposed* RAI*1 as a candidate gene for children with morbid obesity. Furthermore, there were studies which performed NGS in a set of ROHHAD/NET patients [6, 51, 52, 54]. Rand and colleagues [54] analyzed 5-hydroxytryptamine receptor 1A (*HTR1A*), orthopedia (*OTP*), and Adenylate Cyclase Activating Polypeptide 1 (*ADCYAP1*, formerly* PACAP*) genes which are involved in the embryologic development of the hypothalamus and autonomic nervous system in a set of 25 ROHHAD patients and 25 matched controls. Although there were no significantly correlating variations, this report provided evidence that variation of the* HTR1A*,* OTP*, and* ADCYAP1* genes are unlikely responsible for ROHHAD/NET. Barclay et al. [51] analyzed 16 ROHHAD patients using a combination of NGS and Sanger sequencing. They examined mutations in the exons of the genes for hypocretin and accompanying receptors, namely,* HCRT, HCRTR1, *and* HCRTR2,* and found no rare or novel mutations. In this study, we also identified rare variants in two ROHHAD/NET patients. However, the causality of these variants remains unclear and demands further investigation. Nevertheless, we believe that accumulation of these attempts would contribute to progress.

There are some limitations in our research. Firstly, we could not analyze the relationship between the treatments and the subsequent outcomes. Secondly, there remains the possibility of existing case reports or series that were not accessible. Thirdly, some studies only had grouped data where IPD were not available. Nevertheless, this study also has its strengths in that it provides a pooled data and combined evidence on a disease of extreme rarity.

ROHHAD/NET is a rare disease, which has pediatric onset and female preponderance. Rapid obesity and hypothalamic dysfunction are earliest detectable signs. Prompt recognition and timely application of respiratory support may prevent grave complications leading to unprepared mortality. WES on 2 ROHHADNET patients identified no significant mutations or copy number variations. Further analyses of patients in prospective studies are required.

## Figures and Tables

**Table 1:** Summary profiles of individual patient data (IPD) of ROHHADNET syndrome (case-reports).

No.	Patient No.	Authors, year	Age/Sex	Height (cm)/Weight (kg)/BMI	Presenting symptoms	Rapid obesity	Hypothalamic dysfunction	Hypoventilation	Autonomic dysregulation	Behavioral changes	Neurologic findings	Neural crest tumor	Other findings	Na (mmol/L)	Prolactin (ng/mL)	fT4 (ng/dL)	Treatment	Outcome
1	1	Park, 2010 [7]	13/M	161/70.6/28	Pain on both thighs, gait disturbance, general weakness, cold body sensation	No	Yes	Yes	Yes	No	Seizure	Ganglio- neuroma	Rhabdo- myolysis	198	35.8 > 13.	0.5 > 0.8	Hydration, IVIG	Alive

2	2	Thaker, 2015 [12]	2/M	163 (11 yr)/166.3 (11 yr)/62	-	No	No	No	Yes	Yes	No	No	No	Normal	-	1.04	-	Alive

3	3	Gordon, 2015 [13]	4/F	-/-/-	-	No	DI	Sleep apnea	Yes	No	No	Ganglio- neuroblast oma	No	-	Hyper- prolactinemia	Hypo - thyroidism	Caffeine	Alive

4	4	Tellingen, 2015 [14]	4/F	-/-/-	Rapid weight gain, growth retardation	Yes	DI	Yes	No	No	No	Ganglio- neuroma	No	-	-	Hypo - thyroidism	-	Alive

5	5	Grudnikoff, 2013 [15]	4/F	-/-/-	Weight gain, growth retardation, irritability, aggression	Yes	Yes	Sleep apnea	No	Yes	No	Ganglio- neuroma	No	-	-	-	-	Alive

6	6	Patwari, 2011 [16]	8/F	150/45>80/36	-	Yes	Precocious puberty	Sleep apnea	Pupil dilatation	Yes	No	Ganglio- neuroblast oma	Pneumonia, scoliosis	158	56	-	Artificial ventilation	Alive

7	7	Sartori, 2012 [17]	4/M	-/-/-	-	Yes	Polyuria, polydipsia	Sleep apnea	Yes	Yes	No	No	No	Normal	Hyper- prolactinemia	-	IVIG, artificial ventilation	Alive

7	8	Sartori, 2012 [17]	5/F	-/-/-	-	Yes	Yes	Yes	No	Yes	No	No	No	-	-	Central hypo- thyroidism	-	Alive

8	9	Dhondt, 2013 [18]	3/F	-/-/-	Stagnation of neurodevelopment, aggression, hyperphagia	Yes	Precocious puberty	Yes	Yes	Yes	Yes	No	No	-	-	-	Artificial ventilation	Alive

9	10	Bougnères, 2008 [19]	4/-	-/-22	-	Yes	Yes	Yes	Yes	Mental retardation, psychosis	No	Ganglio- neuroma	No	156	19	8.5	-	Alive

9	11	Bougnères, 2008 [19]	3/-	-/-/40	-	Yes	Yes	Sleep apnea	Yes	Mental retardation	No	Ganglio- neuroma	No	161	39	9.8	-	Alive

9	12	Bougnères, 2008 [19]	3/-	-/-/29	-	Yes	Yes	Yes	Yes	No	No	Ganglio- neuroma	No	150	14	17.1	-	Alive

9	13	Bougnères, 2008 [19]	3/-	-/-/35	-	Yes	Yes	Sleep apnea	Yes	No	No	Ganglio- neuroma	No	151	22	16	-	Alive

9	14	Bougnères, 2008 [19]	2/-	-/-/24	-	No	Yes	Yes	Yes	No	No	Ganglio- neuroma	No	145	31	16	-	Alive

9	15	Bougnères, 2008 [19]	2/-	-/-/44	-	No	Yes	Yes	Yes	No	No	Ganglio- neuroma	No	149	34	12.4	-	Alive

10	16	Paz-Priel, 2011 [20]	5/F	-/-/17 > 25	-	Yes	Yes	No	Left exotropia	Aggressive behavior	No	Ganglio- neuroblast oma	No	-	76.5	Normal	Cyclophosphamide, IVIG, prednisone, rituximab	Alive

11	17	Chandrakantan, 2012 [21]	5/F	108/29/25	-	Yes	DI	Yes	Pupil dilatation, pupil response decrease	No	No	Ganglio- neuroblast oma	No	Hyper Na	Hyper- prolactinemia	-	Artificial ventilation, tracheostomy	Alive

11	18	Chandrakantan, 2012 [21]	9/F	137/54/29	-	Yes	DI	Yes	Chronic constipation, neurogenic bladder	No	Developmental delay	No	No	Hyper Na	-	-	Noninvasive mask	Alive

12	19	Kocaay, 2014 [22]	13/F	145/69/32 (10 yrs)	Respiratory distress, cyanosis	Yes	Hypogonadism, secondary amenorrhea, precocious puberty	Yes	No	Social withdrawal	Drowsiness	No	Megaloblastic anemia, acanthosis nigricans, Raynaud phenomenon	151	1.044 (10 yrs)	0.88	-	Alive

13	20	Sumanasena, 2012 [23]	10/F	-/35/-	Progressive respiratory difficulty, edema	Yes	No	Yes	No	Hallucination	Drowsiness	Ganglio- neuroma	No	167	Normal	-	-	Alive

14	21	Abaci, 2013 [3]	3/M	92 > 95.8 (9 mo)/20 > 25.7 (9 mo)/24>28	Cyanosis, recent onset dyspnea	Yes	Polyuria, polydipsia	Yes	No	Yes	No	Ganglione Uroblasto ma	No	143	44.7	0.75	Cyclophosphamide, IVIG, dexamethasone, rituximab	Alive

15	22	Atapattu, 2015 [24]	4/F	-/-/-	Excessive weight gain, increase food seeking, daytime somnolence	Yes	No	Yes	No	Yes	No	Ganglione uroma	Celiac disease	-	-	-	Hypertension medication	Alive

16	23	Uçar, 2013 [8]	6/F	-/-/-	Blurring of consciousness, recurrent fever	No	GH deficiency	Yes	No	No	Yes	Hamartoma tous mass with neural elements	No	152	89	-	Desmopressin acetate, ventilatory support	Alive

17	24	Sethi, 2014 [25]	5/F	117/25 > 37/14>28	Behavior outbursts, poor school performance, hyperphagia, fever, abdominal pain with rectal prolapse	Yes	Yes	No	Bilateral tonic pupils	Yes	No	Ganglione uroblasto ma	Metabolic alkalosis	HypoNa	-	Normal	Endotracheal intubation, risperidone, benzodiazepines, antipsychotic medications	Multiorgan failure, death

18	25	Gallizia, 2012 [26]	3/F	-/-/-	Rapid weight gain, polyuria, sleep apnea	Yes	Yes	Sleep apnea	No	No	No	No	No	-	Hyper- prolactinemia	Normal	-	Alive

18	26	Gallizia, 2012 [26]	3/F	-/-/-	Rapid weight gain, fatigue, polydipsia, syncope episodes, strabismus, behavioral problems	Yes	Yes	No	Strabismus	Yes	Yes	No	No	-	Hyper- prolactinemia	Normal	Mechanical ventilation	Alive

19	27	Baronio, 2013 [27]	1/M	-/-/-	Severe obesity, hyperreninemic hypertension	Yes	No	No	No	No	No	Ganglione uroblasto ma	No	-	-	0.62	Mechanical ventilation, brain hypothermia, steroid pulse	Alive

19	28	Baronio, 2013 [27]	2/F	-/-/-	Severe obesity, hyperreninemic hypertension	Yes	GH deficiency	No	No	No	No	Ganglione uroblasto ma	No	-	Hyper- prolactinemia	0.17	Mechanical ventilation, brain hypothermia, steroid pulse	Alive

20	29	Chow, 2014 [28]	15/M	174/87/29	Fever, headache, vomiting, weight gain	Yes	No	No	No	Irritability, lethargy, somnolence	No	No	No	150>123	-	-	Mechanical ventilation, IVIG, methylprednisolone	Alive

21	30	Kot, 2012 [29]	9/M	short stature/-/-	Weight gain, short stature, hyperphagia, hypodipsia, thermal dysregulation, excessive perspiration, cold extremity, livedo reticularis, sleep apnea	Yes	Hypodipsia, GH deficiency	Sleep apnea	Yes	No	No	No	No	161	Hyper-prolactinemia	Normal	GH replacement	Alive

22	31	Cemeroglu, 2015 [30]	5/-	-/-/-	Short stature, obesity	Yes	DI, GH deficiency	Sleep apnea	Yes	Flat affect	No	No	Scoliosis	157>153	-	0.9>0.5	GH replacement, levothyroxine, desmopressin, tonsillectomy, adenoidectomy, CPAP	Alive

23	32	Chew, 2011 [31]	11/M	-/35/26	Fever, drowsiness, shallow breathing	No	DI, hypogonadism	Sleep apnea	Thermal dysregulation, excessive sweating, right divergent squint	0	Seizure, developmental delay	No	Respiratory acidosis	192	Hyper-prolactinemia	Normal	Anti-epileptics	Alive

24	33	Petty, 2014 [32]	-/M	-/-/-	Weight gain, enuresis, sleep apnea, fever	Yes	No	Yes	Transient visual loss	Hallucination	No	No	Thrombocy topenia	-	-	-	IVIG, CPM, rituximab	Alive

25	34	Maksoud, 2015 [33]	6/F	119/38/27	Abdominal mass, rapid onset obesity	Yes	Premature thelarche, GH deficiency	Sleep apnea	Urinary incontinence	No	No	Ganglione uroma	Hepatitis C	156	-	0.79	-	Alive

26	35	Sanklecha, 2016 [34]	2/F	-/-/-	Gait disturbance, head jerky movement, nystagmus	Yes	DI, polyuria, polydipsia	Yes	No	Aggressiveness	Seizure	Ganglione uroblasto ma	No	189>115	-	-	Chemotherapy, mechanical ventilation, tracheostomy, nasal BIPAP, rituximab, CPM	Cardiac arrest, sudden demise

27	36	Erensoy, 2016 [35]	8/F	-/-/-	Overweight, recession, fatigue, decreased school success	Yes	No	No	No	Poor school performance, MDD, ADHD	No	No	No	-	-	-	Fluoxetine, methylphenidate	Alive

28	37	Al-Harbi, 2016 [36]	8/F	126/45/28	Progressive fatigue, skin bluish discoloration, fever	Yes	Breast enlargement	Shortness of breath, sleep apnea	Cold intolerance, excessive sweating, altered pain sense	Slow mental function, poor school performance, sleepiness	No	No	No	186	Hyper-prolactinemia	Normal	BIPAP	Alive

29	38	Aljabban, 2016 [37]	4/F	110/25/-	Rapid weight gain, excessive eating	Yes	polyuria, polydipsia	Sleep apnea	Cold extremity, GI dysmotility	Mood alteration, anxiety, aggressiveness, recurrent fatigue, social withdrawal, sleepiness	Seizure	Ganglio- neuroma	No	Normal > 162	-	Normal	Antipsychotics, mechanical ventilation, tracheostomy	Cardiac arrest, death

30	39	Bagheri, 2017 [4]	5/F	120/40/-	Cough, cyanosis	Yes	Central hypothyroidism	Sleep apnea	Cold extremities, hyperhidrosis, constipation	Mood change	Seizure	Ganglio- neuroblast oma	Facial plethora, buffalo neck	125	Hyper-prolactinemia	0.8	Mechanical ventilation, tracheostomy	Alive

31	40	Galewicz-zielinska, 2012 [38]	9/-	-/-/-	-	No	No	Mixed sleep apnea	No	Yes	No	No	Tonsillar hypertrophy	-	-	-	BIPAP	Alive

32	41	Jacobson, 2016 [39]	2/F	-/-/40	Hyperphagia, weight gain (16.8 > 35.5 kg)	Yes	Partial DI	Sleep apnea	Reduced pain perception, strabismus	Social withdrawal, autism	No	Ganglio- neuroblast oma	Papular rash	-	-	-	Rituximab, cyclophosphamide	Alive

32	42	Jacobson, 2016 [39]	3/M	-/-/34	Hyperphagia, weight gain (18 kg for 3 mo)	Yes	Yes	Sleep apnea	excessive sweating, thermal dysregulation, enuresis, altered pain sense, strabismus	Social withdrawal	No	No	No	-	Hyper-prolactinemia	-	Rituximab, CPM	Alive

33	43	Lucas-Herald, 2012 [40]	1/F	-/32 (3 yr)/22	Hyperphagia, food stealing	Yes	Hyper-prolactinemia, GH deficiency, water imbalance	Sleep apnea	Altered pain perception	No	No	No	Renal failure	184	-	-	BIPAP	Alive

33	44	Lucas-Herald, 2012 [40]	2/M	-/33/29	Obesity	Yes	Hyper-prolactinemia, failed GH	Mixed sleep apnea	No	No	No	No	No	-	-	-	BIPAP	Alive

34	45	Ibanez-Mico, 2017 [41]	2/F	-/-/-	Obesity, increased appetite	Yes	Hyper-prolactinemia, Central hypothyroidism	Sleep apnea	Yes	Aggression, hyperactivity, impulsivity	Yes	No	Altered pain sense, GI dysmotility	175	166	1.05	IVIG, steroids, cyclophosphamide Tracheostomy	Sudden death

35	46	Isasa, 2018 [42]	10/M	136/66.5/34.92	Seizures (hyponatremia)	Yes	Hyper-prolactinemia Central hypothyroidism	Central hypoventilation Thermal dysregulation	Polydipsia	Aggressiveness	Yes	No	No	Hyper-/hypo-	-	-	-	Alive

36	47	Siraz, 2018 [43]	7/F	130/61/36.0	obesity	Yes	Central hypothyroidism DI, MDD, Central precocious puberty GH deficiency Hyper-prolactinemia Secondary adrenal insufficiency	No	Excessive sweating hypothermia	No	No	No	Pulmonary hyper-tension IQ 65	156	33	0.7	-	Alive

36	48	Siraz, 2018 [43]	5/F	101/31/30.4	Obesity, seizure	Yes	Central hypothyroidism Hyper-prolactinemia	Central hypoventilation	Yes	Aggressiveness	Yes	No	Central cyanosis IQ of 3 years of age	164	56	0.8	Tracheostomy	Alive

ADHD, attention deficit hyperactivity disorder; BIPAP, bilevel positive airway pressure; CPAP, continuous positive airway pressure; CPM, cyclophosphamide; DM, diabetes mellitus; DI, diabetes insipidus; IVIG, intravenous immunoglobulin; IQ, intellectual quotient; GH, growth hormone; GI, gastrointestinal; OCD, obsessive-compulsive disorder; SIADH, syndrome of inappropriate antidiuretic hormone secretion.

**Table 2:** Summary profiles of aggregate patient data of ROHHADNET syndrome (case-series and cohorts).

**Author, year**	**N**°** patients **	**Age (yr)**	**Sex (M/F)**	**Rapid obesity**	**Hypothalamic dysfunction (N**°** patients)**	**Hypoventilation (N**°** patients)**	**Autonomic dysregulation (N**°** patients)**	**Behavioral changes (N**°** patients)**	**Neurologic symptoms (N**°** patients)**	**Neuroendocrine tumors (N**°** patients)**	**Other findings (N**°** patients)**	**Na (mmol/L) (N**°** patients)**	**Treatment (N**°** patients)**	**Outcome (N**°** patients)**
Gil, 2012 [44]	5	-	-	Yes	Yes(5), hypothyroidism(1), adrenal insufficiency(1), precocious puberty(1)	Central apnea(2), transient obstructive apnea(2)	Yes(5)	Yes(2)	No	Ganglio-neuroma (3)	No	-	-	Death(1)

Reppucci, 2014 [45]	7	8.3 (4.7~10.1)	-	Yes	No	Yes(3), sleep apnea(5)	No	No	No	Yes	No	-	-	Alive

Barclay, 2016 [51]	16	4.3	-	No	Yes(16)	Yes(16),	Yes(16)	No	No	Yes(7)	No	-	Artificial ventilation(16)	Alive

Biancheri, 2013 [46]	6	-	2/4	Yes	Hypothyroidism(5), adrenal insufficiency(2), precocious puberty(2)	Central apnea(4)	No	Yes(6)	No	No	No	Electrolyte imbalance (6)	-	Alive

Napoli, 2014 [47]	6	2~4	-	Yes	Hypothyroidism(5), adrenal insufficiency(3), precocious puberty(2)	Central apnea(4)	No	Yes(6)	No	No	No	Electrolyte imbalance (6)	Non-invasive ventilation	Alive

Napoli, 2014 [47]	7	-	-	Yes(7)	Hypothyroidism(6), adrenal insufficiency(4), precocious puberty(2)	Sleep apnea(7)	No	Yes(7)	No	Ganglio-neuroma (3)	No	-	-	Alive

Ize-Ludlow, 2007 [6]	15	-	6/9	Yes(8)	Hypothyroidism(5), adrenal insufficiency(4), precocious puberty(2), delayed puberty(2), amenorrhea(1), irregular menstruation(1), premature adrenarche(2), hypogonadism(1), SIADH(2), polydipsia(8), hypodipsia(4), polyuria(4)	Alveolar hypoventilation (15). sleep apnea(8), cyanosis(4)	Ophthalmologic manifestations (13), thermal dysregulation (11), GI dysmotility(10), Altered pain perception(8), altered sweating(8), cold extremity(6)	Depression(2), flat effect(2), psychosis(2), behavioral outbursts(1), bipolar disorder(1), emotional lability(1), OCD(1), oppositional-defiant disorder(1), Tourette's syndrome(1), hallucination(1)	Syncope(1), developmental delay(3), regression(3), seizure(5), hypotonia (4)	Yes(5)	Scoliosis(3), type 2 DM(2), enuresis(4), asthma(3), hyper-somnolence(2), pneumonia(2)	HyperNa (7), hypoNa (2)	-	Cardiac arrest(9)

Barclay, 2015 [48]	35	-	14/21	Yes	Yes(35)	Yes(35)	Yes(35)	No	No	Yes(15)	No	-	Artificial ventilation (35)	Alive

Gueorguieva, 2011 [49]	9	0~4	-	No	Hypogonadism(4)	Yes(9)	Yes(9)	Mental retardation(4)	No	Ganglio-neuroma (6)	No	Mean 150	-	Death (2)

Abel, 2010 [50]	4	-	1/3	Yes	No	Alveolar hypoventilation	Thermal dysregulation, cold extremity, altered pain perception	Emotional lability, behavioral outburst	No	No	No	-	-	Alive

**Table 3:** Clinical presentations of patients with ROHHADNET syndrome (IPD).

**Clinical findings**	**Total number of patients (n=48)**
	**Number of patients (**%**)**
**Rapid obesity **	**40 (83.3**%**)**
**Hypoventilation**	**36 (75.0**%**)**
Obstructive sleep apnea	21 (43.8%)
Respiratory distress	5 (10.4%)
Cyanotic episodes	4 (8.3%)
**Hypothalamic dysfunction**	**40 (83.3**%**)**
Growth hormone deficiency	13 (25.3%)
Diabetes insipidus	9 (18.8%)
Polyuria/polydipsia	8 (16.7%)
Central precocious puberty	7 (14.6%)
Hypogonadotropic hypogonadism	2 (4.2%)
Premature thelarche	2 (4.2%)
**Autonomic dysregulation**	**32 (66.7**%**)**
Ophthalmologic abnormality	12 (25.0%)
Altered perception of pain	6 (12.5%)
Gastrointestinal dysmotility	6 (12.5%)
Cold extremity	4 (8.3%)
Neurogenic bladder	4 (8.3%)
Excessive sweating	5 (10.4%)
Thermal dysregulation	3 (6.3%)
Syncope	1 (2.1%)
Urinary incontinence	1 (2.1%)
**Behavioral disorders**	**29 (60.4**%**)**
Irritability & aggression	10 (20.8%)
Fatigue	4 (8.3%)
Social withdrawal	4 (8.3%)
Poor school performance	3 (6.3%)
Intellectual disability	2 (4.2%)
Mood change	2 (4.2%)
Flat affect	2 (4.2%)
Hallucination	2 (4.2%)
Major depressive disorder	1(2.1%)
Attention deficit disorder	1(2.1%)
Psychosis	1(2.1%)
**Neurologic abnormality**	**16 (33.3**%**)**
Seizure	7 (14.6%)
Blurring of consciousness	4 (8.3%)
Sleep disturbance	3 (6.3%)
Developmental delay	3 (6.3%)
Gait disturbance	2 (4.2%)
Nystagmus	1 (2.1%)
General weakness	1 (2.1%)
**Other findings**	
Fever	6 (12.5%)
Papular rash	3 (6.3%)
Enuresis	2 (4.2%)
Scoliosis	2 (4.2%)
Rhabdomyolysis	1 (2.1%)
Pneumonia	1 (2.1%)
Headache	1 (2.1%)
Megaloblastic anemia	1 (2.1%)
Thrombocytopenia	1 (2.1%)
Acanthosis nigricans	1 (2.1%)
Raynaud phenomenon	1 (2.1%)
Celiac disease	1 (2.1%)
Metabolic alkalosis	1 (2.1%)
Hepatitis C	1 (2.1%)
Buffalo neck	1 (2.1%)
Tonsillar hypertrophy	1 (2.1%)
Abdominal mass	1 (2.1%)
Renal failure	1 (2.1%)
Edema	1 (2.1%)
Pulmonary hypertension	1 (2.1%)
Cough	1 (2.1%)

**Table 4:** Laboratory findings of patients with ROHHADNET syndrome (IPD).

**Laboratory findings**	**Total number of patients (n=48)**
	**Number of patients (**%**)**
**ABGA **	
Hypoxemia^†^	13/13(100%)
Hypercapnia^‡^	14/15 (93.3%)
Normal	0/15 (0%)
No information	34/48 (70.8%)
**Dysnatremia**	
Hypernatremia	25/31 (80.6%)
Hyponatremia	5/31 (16.1%)
Normal	2/31 (6.5%)
No information	17/48(35.4%)
**Prolactin**	
Hyperprolactinemia	27/28 (96.4%)
Normal	1/28 (3.6%)
No information	19/48 (39.6%)
**Thyroid dysfunction **	
Hypothyroidism	18/30 (60.0%)
Normal	12/30 (40.0%)
No information	17/48 (35.4%)
**IGF-1 level**	
Low	12/16 (75.0%)
Normal	4/16 (25.0%)
No information	31/48 (64.6%)

ABGA: Arterial blood gas analysis, IGF-1: Insulin-like growth factor-1.

^†^Hypoxemia is defined in terms of reduced partial pressure of oxygen below 80 mmHg or decreased oxygen saturation less than 90%.

^‡^Hypercapnia is defined in terms of elevated carbon dioxide above 45 mmHg.

**Table 5:** Treatment of case-reported patients with ROHHADNET syndrome (IPD).

**Treatment**	**Total number of patients (n = 48)**
	**Number of patients (**%**)**
**Respiratory support**	**21 (43.8**%**)**
Mechanical ventilation	20 (41.7%)
BIPAP	7 (14.6%)
CPAP	1 (2.1%)
Noninvasive mask	1 (2.1%)
Tracheostomy	6 (12.5%)
**Steroids**	**7 (14.6**%**)**
Methylprednisolone	2 (4.2%)
Steroid pulse therapy	2 (4.2%)
Prednisolone	2 (2.1%)
Dexamethasone	1 (2.1%)
**Fluid resuscitation**	**4 (8.3**%**)**
**Intravenous immunoglobulins**	**7 (14.6**%**)**
**Immunosuppressive agents**	**7 (14.6**%**)**
Rituximab	5 (10.4%)
Cyclophosphamide	6 (12.5%)
**Other agents **	
Antipsychotics	3 (6.3%)
Desmopressin acetate	2 (4.2%)
GH replacement	2 (4.2%)
Anti-epileptics	2 (4.2%)
Levothyroxine	4 (8.3%)
Caffeine	1 (2.1%)
Hypertensive medication	1 (2.1%)
**Procedure**	**2 (4.2**%**)**
Brain hypothermia	1 (2.1%)
Tonsillectomy	1 (2.1%)

BIPAP: bilevel positive airway pressure; CPAP: Continuous Positive Airway Pressure; GH: growth hormone.

**Table 6:** Tumor presentation of patients with ROHHADNET syndrome (IPD and aggregate data).

**Author, year [ref] **	**Type/histology**	**Location/size**	**Associated symptoms/signs**	**Treatment**
Park, 2010 [7]	Ganglioneuroma	Right adrenal	N/A	IVIG

Gordon, 2015 [13]	Ganglioneuroblastoma	Left adrenal	N/A	N/A

Tellingen, 2015 [14]	Ganglioneuroma	N/A	N/A	N/A

Grudnikoff, 2013 [15]	Ganglioneuroma	N/A	N/A	resection

Patwari, 2011 [16]	Ganglioneuroblastoma	Right paraspinal	N/A	N/A

Bougnères, 2008 [19]	Ganglioneuroma (6 patients)	1 mediastinal 2 right adrenal 3 left adrenal	N/A	N/A

Paz-Priel, 2011 [20]	Ganglioneuroblastoma	Retroperitoneal mass	Opsoclonus- myoclonus-ataxia syndrome	resection, cyclophosphamide, IVIG

Chandrakantan, 2012 [21]	Ganglioneuroblastoma	Left adrenal	N/A	resection

Sumanasena, 2012 [23]	Ganglioneuroma	Left adrenal	N/A	resection

Abaci, 2013 [3]	Ganglioneuroma/ intermixed type with favorable histology	Retroperitoneal mass (6.5 × 3.5 × 2.0 cm)	N/A	Resection, cyclophosphamide, IVIG, dexamethasone

Atapattu, 2015 [24]	Ganglioneuroma	Right adrenal	N/A	resection

Ucar, 2013 [8]	Hamartomatous mass with neural elements of benign nature	Parahilar mass (2.5 cm)	N/A	Resection

Sethi, 2014 [25]	Ganglioneuroblastoma	Right adrenal mass (4.0 × 3.0 × 4.0 cm)	N/A	N/A

Baronio, 2013 [27]	Ganglioneuroblastoma intermixed	N/A	Hypertension, Cushing syndrome	Resection

Maksoud, 2015 [33]	Ganglioneuroma	Paravertebral mass (8.0 × 3.5cm) compressing the right ureter	Right hydroureteronephrosis	Resection

Sanklecha, 2016 [34]	Ganglioneuroblastoma	Paravertebral mass	Gait disturbance	Resection, chemotherapy (not specified)

Aljabban, 2016 [37]	Ganglioneuroma	Posterior mediastinal mass (10 × 10 cm)	N/A	resection

Bagheri, 2017 [4]	Ganglioneuroblastoma	Mediastinal mass (1.5 cm)	N/A	N/A

Jacobson, 2016 [39]	Ganglioneuroma	N/A	N/A	resection

IVIG, intravenous immunoglobulin; N/A, not available for information.

**Table 7:** Reported human candidate genes for ROHHAD/NET.

**Gene **	**Location **	**Protein**	**Function **	**Reference Number **
*RAI1*	17p11.2	p.R1089X	Craniofacial and nervous system development	Thaker et al. [12]

*NTRK2*	9q21.33	p.P204H Tropomyosin receptor kinase B (TrkB),	Neuroendocrine /synaptic plasticity	Ize-Ludlow et al. [6]

*NECDIN*	15q11–q13	Necdin (p.V318A)	Hypothalamic/respiratory	De Pontual et al.[52]

*ASCL1*	12q23.2	Human achaete-scute homolog 1 (hASH1)	Neuroendocrine	De Pontual et al. [52]

*PHOX2B*	4p13,	Paired mesoderm homeobox protein 2B (NBPhox)	Respiratory/autonomic	Ize-Ludlow et al. [6] De Pontual et al. [52]

*BDNF*	11p14.1	Brain-derived neurotrophic factor (BDNF)	Neuronal development/synaptic plasticity	Ize-Ludlow et al.[6] Han et al. [53]

*HCRT *	17q21.2	Hypocretins	Sleep/wake regulation, energy balance, and the control of breathing	Barclay et al. [51]

*HCRTR1*	1p35.2	Hypocretin receptor type 1 (HcrtR1),	Sleep/wake regulation, energy balance, and the control of breathing	Barclay et al. [51]

*HCRTR2*	6p12.1	Hypocretin receptor type 2 (HcrtR2),	Sleep/wake regulation, energy balance, and the control of breathing	Barclay et al. [51]

*HTR*_*1A*_	5q12.3	5-hydroxytryptamine (serotonin) receptor 1A	Appetite control, energy regulation, autonomic response to homeostatic stress	Rand et al. [54]

*OTP*	5q14.1	Orthopedia (Otp) homeodomain protein	Hypothalamic expression, with an important role in hypothalamic cell specification in the developing hypothalamus	Rand et al. [54]

*ADCYAP1*	18p11.32	Adenylate Cyclase Activating Polypeptide 1	Maintenance of normal energy homeostasis, respiratory chemosensitivity and preventing neonatal hypoventilation at reduced body temperatures	Rand et al. [54]

## Data Availability

The data used to support the findings of this study are included within the main manuscript and the supplementary information file.

## Supplementary Materials

Supplementary Materials Supplementary Table S1: checklist summarizing compliance with PRISMA guidelines Supplementary Table S2: clinical details of the two patients included in WES. Supplementary Table S3: filtering process of whole exome sequencing analysis performed in two patients. Supplementary Table S4: possible variants identified in individuals with ROHHAD syndrome by WES.
