# Supplementary material for: Rapid-Onset Obesity with Hypoventilation, Hypothalamic, Autonomic Dysregulation, and Neuroendocrine Tumors (ROHHADNET) Syndrome: A Systematic Review
Source: Biomed Res Int. 2018 Nov 21;2018:1250721. doi: 10.1155/2018/1250721 (PMC6280256; doi:10.1155/2018/1250721)
Supplement: Supplementary Materials — Supplementary Table S1: checklist summarizing compliance with PRISMA guidelines Supplementary Table S2: clinical details of the two patients included in WES. Supplementary Table S3: filtering process of whole exome sequencing analysis performed in two patients. Supplementary Table S4: possible variants identified in individuals with ROHHAD syndrome by WES. [file 1250721.f1.docx]

**Supplementary Tables**

**Rapid-onset obesity with hypoventilation, hypothalamic, autonomic dysregulation and neuroendocrine tumors**

**(ROHHADNET) syndrome: A systematic review**

Jiwon M. Lee^1,^*, Jaewon Shin^2,^*, Sol Kim^3,^*, Heon Yung Gee^4^, Joon Suk Lee^4^, Do Hyeon Cha^4^, John Hoon Rim^4^, Se-Jin Park^5^, Ji Hong Kim^2^, Ahmet Uçar^6^, Andreas Kronbichler^7^, Keum Hwa Lee^2,8^, and Jae Il Shin ^2,8,9^

^1^Department of Pediatrics, Chungnam National University Hospital, Daejeon, Republic of Korea

^2^Department of Pediatrics, Yonsei University College of Medicine, Seoul, Republic of Korea

^3^Yonsei University College of Medicine, Seoul, Republic of Korea

^4^Department of Pharmacology, Yonsei University College of Medicine, Seoul, Republic of Korea

^5^Department of Pediatrics, Geoje Children's Hospital, Ajou University School of Medicine, Geoje, Republic of Korea

^6^Department of Pediatric Endocrinology and Diabetes, Sisli Etfal Training and Research Hospital, Istanbul, Turkey

^7^Department of Internal Medicine IV (Nephrology and Hypertension), Medical University Innsbruck, Innsbruck, Austria

^8^Department of Pediatric Nephrology, Severance Children’s Hospital, Seoul, Republic of Korea

^9^Institute of Kidney Disease Research, Yonsei University College of Medicine, Seoul, Republic of Korea

**Correspondence:** Jae Il Shin, M.D., Ph.D.

Address: 50 Yonsei-ro, Seodaemun-gu, C.P.O. Box 8044, Department of Pediatrics, Yonsei University College of Medicine, Seoul 120-752, Korea

Tel. +82-2-2228-2050; Fax. +82-2-393-9118; E-mail: shinji@yuhs.ac

*These authors contributed equally to the work

**Supplementary Table S1.** Checklist summarizing compliance with PRISMA guidelines

| **Section/topic** | **#** | **Checklist item** | **Reported on page #** |
| --- | --- | --- | --- |
| **TITLE** | | |  |
| Title | 1 | Identify the report as a systematic review, meta-analysis, or both. | Title |
| **ABSTRACT** | | |  |
| Structured summary | 2 | Provide a structured summary including, as applicable: background; objectives; data sources; study eligibility criteria, participants, and interventions; study appraisal and synthesis methods; results; limitations; conclusions and implications of key findings; systematic review registration number. | 2-3 |
| **INTRODUCTION** | | |  |
| Rationale | 3 | Describe the rationale for the review in the context of what is already known. | 4 |
| Objectives | 4 | Provide an explicit statement of questions being addressed with reference to participants, interventions, comparisons, outcomes, and study design (PICOS). | 4 |
| **METHODS** | | |  |
| Protocol and registration | 5 | Indicate if a review protocol exists, if and where it can be accessed (e.g., Web address), and, if available, provide registration information including registration number. | N/A |
| Eligibility criteria | 6 | Specify study characteristics (e.g., PICOS, length of follow-up) and report characteristics (e.g., years considered, language, publication status) used as criteria for eligibility, giving rationale. | 5-8 |
| Information sources | 7 | Describe all information sources (e.g., databases with dates of coverage, contact with study authors to identify additional studies) in the search and date last searched. | 5-8 |
| Search | 8 | Present full electronic search strategy for at least one database, including any limits used, such that it could be repeated. | 5-8 |
| Study selection | 9 | State the process for selecting studies (i.e., screening, eligibility, included in systematic review, and, if applicable, included in the meta-analysis). | 5-8 (Figure1) |
| Data collection process | 10 | Describe method of data extraction from reports (e.g., piloted forms, independently, in duplicate) and any processes for obtaining and confirming data from investigators. | 5-8 |
| Data items | 11 | List and define all variables for which data were sought (e.g., PICOS, funding sources) and any assumptions and simplifications made. | 5-8 |
| Risk of bias in individual studies | 12 | Describe methods used for assessing risk of bias of individual studies (including specification of whether this was done at the study or outcome level), and how this information is to be used in any data synthesis. | N/A |
| Summary measures | 13 | State the principal summary measures (e.g., risk ratio, difference in means). | N/A |
| Synthesis of results | 14 | Describe the methods of handling data and combining results of studies, if done, including measures of consistency (e.g., I^2^) for each meta-analysis. | N/A |

| **Section/topic** | **#** | **Checklist item** | **Reported on page #** |
| --- | --- | --- | --- |
| Risk of bias across studies | 15 | Specify any assessment of risk of bias that may affect the cumulative evidence (e.g., publication bias, selective reporting within studies). | N/A |
| Additional analyses | 16 | Describe methods of additional analyses (e.g., sensitivity or subgroup analyses, meta-regression), if done, indicating which were pre-specified. | N/A |
| **RESULTS** | | |  |
| Study selection | 17 | Give numbers of studies screened, assessed for eligibility, and included in the review, with reasons for exclusions at each stage, ideally with a flow diagram. | 9 |
| Study characteristics | 18 | For each study, present characteristics for which data were extracted (e.g., study size, PICOS, follow-up period) and provide the citations. | Supplementary tables |
| Risk of bias within studies | 19 | Present data on risk of bias of each study and, if available, any outcome level assessment (see item 12). | N/A |
| Results of individual studies | 20 | For all outcomes considered (benefits or harms), present, for each study: (a) simple summary data for each intervention group (b) effect estimates and confidence intervals, ideally with a forest plot. | Supplementary tables |
| Synthesis of results | 21 | Present results of each meta-analysis done, including confidence intervals and measures of consistency. | N/A |
| Risk of bias across studies | 22 | Present results of any assessment of risk of bias across studies (see Item 15). | N/A |
| Additional analysis | 23 | Give results of additional analyses, if done (e.g., sensitivity or subgroup analyses, meta-regression [see Item 16]). | N/A |
| **DISCUSSION** | | |  |
| Summary of evidence | 24 | Summarize the main findings including the strength of evidence for each main outcome; consider their relevance to key groups (e.g., healthcare providers, users, and policy makers). | 13-15 |
| Limitations | 25 | Discuss limitations at study and outcome level (e.g., risk of bias), and at review-level (e.g., incomplete retrieval of identified research, reporting bias). | 15 |
| Conclusions | 26 | Provide a general interpretation of the results in the context of other evidence, and implications for future research. | 15 |
| **FUNDING** | | |  |
| Funding | 27 | Describe sources of funding for the systematic review and other support (e.g., supply of data); role of funders for the systematic review. | 15 |

*From:*  Moher D, Liberati A, Tetzlaff J, Altman DG, The PRISMA Group (2009). Preferred Reporting Items for Systematic Reviews and Meta-Analyses: The PRISMA Statement. PLoS Med 6(6): e1000097. doi:10.1371/journal.pmed1000097

**Supplementary Table S2.** **Clinical details of the two patients included in WES.**

| **Individual** | **Case 1 (YPN01)** | | **Case 2(YPN3-21)** |
| --- | --- | --- | --- |
| Gender/age | M/15 | | F/5 |
| Ethinicity | Korean | | Turkish |
| Rapid-onset obesity | | Present | Present (50 pounds in 5-6months) |
| Hypoventilation | | Present | Present |
| Other clinical manifestation | | Rhabdomyolysis,  hypothermia | Hallucinations, attention deficits, bouts of anger, hypoventilation |
| Laboratory abnormality | | Hypernatremia (193mEql/L) | Hypernatremia (153mEq/L) |
|  | |  |  |
| Neuroendocrine tumor | | Right adrenal mass | A parahilar mass (2.5cm) |
|  | | Ganglioneuroma | Hamartomatous mass with neural elements of benign nature |
| *PHOX2B* analysis | | Normal | Normal |

**Supplementary Table S3.** **Filtering process of whole exome sequencing analysis performed in two patients.**

| **Individual** |  | **Case 1 (YPN01)** | **Case 2(YPN3-21)** |
| --- | --- | --- | --- |
| Total sequence reads |  | 67,616,566 | 155,296,948 |
| Matched Reads |  | 67,076,878 (99.20%) | 154,525,627 (99.50%) |
| Total number of variants detected |  | 188,415 | 201,002 |
| Variants which are not common dbSNP138 (MAF>1%) (A) |  | 48,659 | 37,558 |
| Variants filtered by 59 control data |  | 34,204 | 22,275 |
| Variants which are nonsynonymous or located in splice junction (B) |  | 1,914 | 1,282 |
| % B/A |  | 3.93% | 3.41% |
| Located within splice site |  | 116 | 82 |
| Deletion/Insertion |  | 135 | 132 |
| Stop codon gained / Stop codon lost |  | 40 | 18 |
| Missense |  | 1,623 | 1,050 |

**Supplementary Table S4. Possible variants identified in individuals with ROHHAD syndrome by WES.**

| **Family #** | **Gene symbol** | **Nucleotide change****^a^** | **Amino acid change** | **Amino acid sequence conservation****^b^** | **Frequencies in the**  **dbSNP database****^c^** | **Frequencies in the gnomAD database****^d^** | **Mutation Taster****^e^** | **PP2 Humvar****^f^** | **SIFT****^g^** | **Condel****^h^** | **CADD****^i^** |
| --- | --- | --- | --- | --- | --- | --- | --- | --- | --- | --- | --- |
| Case 1 (YPN01) | *PIK3R3* | c.1163A>G | p.Lys388Arg | *D. rerio* | No | 0.000008  (no hom) | DC (1) | Bn (0.007) | Tol (0.51) | Neu (0.009) | 15.4 |
|  |  | c.152A>G | p.Asn51Ser | *M. musculus* | rs558585498 C=0.0003/33 (ExAC) C=0.0002/1 (1000G) | 0.000217  (no hom) | DC (0.976) | Bn (0.022) | Tol (0.19) | Neu (0.051) | 8.255 |
|  | *SPTBN5* | c.10171C>T | p.Arg3391Trp | *X. tropicalis* | rs777214710 A=0.00003/3 (ExAC) A=0.00003/1 (TOPMED) | 0.000021  (no hom) | PM (1) | Bn (0.003) | Tol (0.1) | Neu (0.286) | 12.7 |
|  |  | c.6976G>A | p.Glu2326Lys | *D. rerio* | rs201067348 T=0.0002/29 (ExAC) T=0.0002/1 (1000G) T=0.0002/2 (GO-ESP) T=0.00003/1 (TOPMED) | 0.000209  (no hom) | PM (0.991) | Dam (0.85) | Del (0.01) | Del (0.733) | 23.9 |
|  | *PCF11* | c.1345A>G | p.Ile449Val | *M. musculus* | No | 0.000004  (no hom) | PM (0.998) | Bn (0) | Tol (0.96) | Neu (0.000) | 0.222 |
|  |  | c.1564C>T | p.Arg522Cys | *D. rerio* | rs780673773 T=0.000008/1 (ExAC) | 0.000008  (no hom) | DC (1) | Dam (0.996) | Del (0) | Del (0.906) | 32 |
| Case 2 (YPN3-21) | *SRMS* | c.1225G>A | p.Val409Ile | *D. rerio* | rs372304332  T=0.0002/17 (ExAC) T=0.0006/3 (1000G) | 0.000184  (no hom) | DC (0.961) | Dam (0.94) | Del (0.03) | Del (0.750) | 25.7 |
|  |  | c.173C>T | p.Ala58Val | *G. gallus* | rs771219616 A=0.00005/5 (ExAC) | 0.000025  (no hom) | DC (0.928) | Bn (0.22) | Tol (0.06) | Neu (0.344) | 22.3 |
|  | *ZNF83* | c.839A>T | p.Gln280Leu | *X. tropicalis* | rs200250390 A=0.00002/2 (ExAC) A=0.1741/872 (1000G) | 0.000009  (no hom) | PM (1) | Bn (0) | Del (0.05) | Neu (0.345) | 10.5 |
|  | *KMT2B* | c.6760C>A | p.Pro2254Thr | *M. musculus* | rs763536557 | 0.000071  (no hom) | PM (1) | Bn (0.107) | Tol (0.19) | Neu (0.058) | 19.66 |

Abbreviation are as follows: Bn, benign; CADD, combined annotation dependent depletion score; Condel, consensus deleteriousness score of non-synonymous single nucleotide variants; Dam, damaging; DC, disease causing; Del, deleterious; Neu, neutral; PM, polymorphism; PP2, PolyPhen-2 prediction score Humvar; SIFT, sorting Intolerant from Tolerant; SNP, single nucleotide polymorphism; Tol, tolerant.

**^a^**cDNA mutations are numbered according to human cDNA reference sequence NM_001114172.1 (*PIK3R3*), NM_016642.2 (*SPTBN5*), NM_015885.3 (*PCF11*), NM_080823.2 (*SRMS*), NM_001105549.1 (*ZNF83*), and NM_014727.1 (*KMT2B*); +1 corresponds to the A of ATG translation initiation codon. ^b^Amino acid residue is continually conserved throughout evolution including the species as indicated. ^c^dbSNP database (<http://www.ncbi.nlm.nih.gov/SNP>). ^d^gnomAD browser (<http://gnomad.broadinstitute.org/>). ^e^Mutation taster (<http://www.mutationtaster.org/>). ^f^PolyPhen-2 prediction score HumVar ranges from 0 to 1.0; 0 = benign, 1.0 = probably damaging (http://genetics.bwh.harvard.edu/pph2/). ^g^SIFT (<http://sift.jcvi.org/>). ^h^Condel (<http://bbglab.irbbarcelona.org/fannsdb/>). ^i^CADD (<http://cadd.gs.washington.edu/>).
